# Abnormal arginine synthesis confers worse prognosis in patients with middle third gastric cancer

**DOI:** 10.1186/s12935-023-03200-5

**Published:** 2024-01-03

**Authors:** Lianlian Hong, Xi Tang, Jing Han, Jiaqi Wang, Qianqian Xu, Xin Zhu

**Affiliations:** 1 Experimental Research Centre, Hangzhou Institute of Medicine (HIM), Zhejiang Cancer Hospital, Chinese Academy of Science, Hangzhou, China; 2 Key Laboratory of Head & Neck Cancer Translational Research of Zhejiang Province, Zhejiang Cancer Hospital, Hangzhou, China; 3 Biological Sample Bank, Hangzhou Institute of Medicine (HIM), Zhejiang Cancer Hospital, Chinese Academy of Science, Hangzhou, China; 4 Postgraduate training base Alliance of Wenzhou Medical University (Zhejiang Cancer Hospital), Hangzhou, China

## Abstract

**Background:**

Gastric cancer at different locations has distinct prognoses and biological behaviors, but the specific mechanism is unclear.

**Methods:**

Non-targeted metabolomics was performed to examine the differential metabolite phenotypes that may be associated with the effects of tumor location on the prognosis of gastric cancer. And silencing of the rate-limiting enzyme to evaluate the effect of abnormal changes in metabolic pathway on the functional biological assays of gastric cancer cells HGC-27 and MKN28.

**Results:**

In a retrospective study of 94 gastric cancer patients, the average survival time of patients with gastric cancer in the middle third of the stomach was significantly lower than that of patients with gastric cancer in other locations (*p* < 0.05). The middle third location was also found to be an independent risk factor for poor prognosis (HR = 2.723, 95%CI 1.334–5.520), which was closely associated with larger tumors in this location. Non-targeted metabolomic analysis showed that the differential metabolites affected 16 signaling pathways including arginine synthesis, retrograde endocannabinoid signaling, arginine biosynthesis, and alanine and aspartate and glutamate metabolism between gastric cancer and normal tissue, as well as between tumors located in the middle third of the stomach and other locations. Argininosuccinate synthetase 1 (ASS1), the rate-limiting enzyme of the arginine biosynthesis pathway, catalyzes the production of argininosuccinic acid. Here, knockdown of ASS1 significantly inhibited the proliferation, colony formation, and migration/invasion of gastric cancer cells, and promoted apoptosis.

**Conclusions:**

Our study suggests that abnormal arginine synthesis may lead to larger tumor size and worse prognosis in gastric cancer located in the middle third position of the stomach. These findings may provide the basis for the stratification and targeted treatment of gastric cancer in different locations.

**Supplementary Information:**

The online version contains supplementary material available at 10.1186/s12935-023-03200-5.

## Introduction

In 2020, the incidence and mortality associated with gastric cancer ranked fifth and fourth worldwide, respectively, among malignant tumors [1]. China has one of the highest incidences of gastric cancer. In 2020, there were 480,000 new cases of gastric cancer in China, accounting for 44% of new cases worldwide. Furthermore, there were 370,000 gastric cancer-related deaths in China, representing nearly half of global gastric cancer-associated fatalities [2].

According to the “Gastric Cancer Treatment Regulations” formulated by the Japanese Association for Gastric Cancer Research, the stomach can be divided into three parts, the upper third (proximal), middle third and lower third (distal) segments, using lines across three equal points between the greater curvature and lesser curvature of the stomach as the boundary [3]. Although the association between tumor location and progression or prognosis of gastric cancer has been extensively studied, it remains a controversial issue [4–6]. It is widely believed that gastric cancer in different anatomical locations exhibits distinct clinicopathological features and biological behaviors; however, the impact of tumor location on the prognosis of gastric cancer has yet to be determined.

Metabolic irregularities play a crucial role in cancer development. Metabolomics offers a means to collect low-molecular-weight metabolites and investigate the subsequent gene and protein products [7]. Metabolomics also serves as a powerful tool for early diagnosis, prognosis assessment, and evaluation of drug efficacy. Metabolomics has been utilized in gastric cancer research, specifically in the identification of biomarkers and therapeutic targets [7–10]. Furthermore, metabolomics has allowed the challenging issue of peritoneal metastasis in gastric cancer to be addressed [11]. To summarize, the development and progression of gastric cancer are closely linked to metabolic disorders and aberrant alterations in associated metabolic profiles.

Our present retrospective study revealed that patients with gastric cancer located in the middle third segment of the stomach (middle third gastric cancer) had a worse prognosis than patients with gastric cancer at other locations. Furthermore, middle third gastric cancer was found to be an independent risk factor for poorer prognosis. Using non-targeted metabolomics analysis, we depicted the characteristic metabolic profile of middle third gastric cancer, specifically, abnormal changes in the arginine synthesis pathway. Knockdown of the key rate-limiting enzyme argininosuccinate synthetase 1 (ASS1) of the arginine synthesis pathway was found to inhibit colony formation, proliferation and migration of gastric cancer cells. Our findings contribute to the stratification of gastric cancer patients based on tumor location, and ultimately facilitate the development of personalized and targeted therapeutic approaches for gastric cancer.

## Materials and methods

### Cell Culture

Human gastric cancer cell lines, MKN28 (RRID:CVCL_1416) and HGC-27 (RRID:CVCL_1279), were purchased from the Shanghai Cell Bank of the Chinese Academy of Sciences. The cell lines were cultured in 1640 medium (Gibco, USA) containing 10% fetal bovine serum (FBS, Gibco, USA).

### Patient clinical data

The pathological and prognostic data of 94 gastric cancer patients admitted to Zhejiang Cancer Hospital between 2010 and 2017 were collected. The surgical approach was radical gastrectomy, and all patients underwent D2 lymph node dissection with negative surgical margins. Postoperative pathological diagnosis was performed by the Department of Pathology at the hospital. Inclusion criteria included: pathologically diagnosed primary gastric cancer, no history of neoadjuvant chemoradiotherapy, no diagnosis of other cancers, and no surgical history of other stomach diseases. Patients’ medical data, including gender, age, surgical method, tumor size, tumor location, histological type, T stage, N stage, positive rate of lymph nodes, and history of adjuvant therapy were retrieved from the medical record system of the hospital. The start time of follow-up was defined as the date of surgery, and the end point of follow-up was the time of death or the time of last follow-up. Follow-up ended in June 2020.

According to the guidelines issued by the Japanese Gastric Cancer Research Association, patients were separated into four groups: Upper third, Middle third, and Lower third, as well as a Mixed group, which included cases that were difficult to clearly define. Postoperative pathological diagnosis was made based on the tumor-node-metastasis (TNM) staging system of the 8th edition of UICC/AJCC in 2016.

### Samples for metabolomics analysis

Non-targeted metabolomics analysis was carried out in 23 gastric adenocarcinoma patients who had been surgically resected and diagnosed by a pathological exam in Zhejiang Cancer Hospital between May 2010 and April 2020. The average age of the patients was 58.22 years old, and the male to female ratio was 19:4. All patients were stage III, and included 5 Upper third, 7 Middle third and 11 Lower third cases. Tumor tissue and normal tissue samples were collected from each patient. The sample data are shown in Additional file 1: Table S1. T (tumor tissues) versus (vs.) N (normal tissues) group and Middle (middle third gastric cancer) vs. Upper/Lower (upper/lower third gastric cancer) group were compared.

### Extraction of metabolites, liquid chromatography-mass spectrometry (LC-MS) analysis, metabolomics data analysis

Please refer to Additional file 2: Appendix S1 for detailed experimental methods.

### siRNA knockdown assay

The siRNA sequences of the *ASS1* gene (si-ASS1, Gemma Biotechnology Co., Ltd., CHINA) were listed in Additional file 3: Table S2. Cells were transfected with si-ASS1 using Lipofectamine 3000 (Invitrogen, USA) according to the manufacturer’s instructions.

### Western blotting

Total protein was extracted from cells using RIPA lysis buffer containing protease inhibitors (Beyotime, CHINA), and quantified using the BCA protein assay (Beyotime, CHINA). Proteins were separated by 12% SDS-PAGE, transferred to a 0.45 μm PVDF membrane, blocked with 5% skimmed milk at room temperature for 2 h, and then incubated with the antibody against β-actin (RRID: AB_2943481) or ASS1 (RRID: AB_2943482) (Huabio, CHINA) at 4 °C overnight. The following day, membranes were incubated for 1 h with HRP-conjugated secondary antibody (Huabio, CHINA), washed, and developed with ECL Plus (Amersham Pharmacia Biotech, UK).

### Colony formation assay

Control and si-ASS1-transfected HGC-27 and MKN28 cells were seeded into 6-well plates at a density of 1 × 10^3^ per well and cultured in an incubator for 7–14 days. The colonies were washed with PBS, and fixed with 4% paraformaldehyde (Beyotime, CHINA) at room temperature for 20 min, then stained with 0.5% crystal violet (Beyotime, CHINA) for 20 min. After washing and drying, cell colonies were counted using ImageJ software. The experiment was repeated three times.

### Cell proliferation assay

Control and si-ASS1-transfected HGC-27 and MKN28 cells were seeded into 96-well plates at a density of 2 × 10^3^ per well. Cells were incubated with 100 µL of culture medium containing 10% CCK8 (DOJINDO, Japan) for 2 h. The absorbance was read at 450 nm with a microplate reader. The experiment was repeated three times.

### Migration assay

Control and si-ASS1-transfected HGC-27 and MKN28 cells were seeded into the upper compartment of the transwell chamber (Corning, USA) in serum-free media. Complete media containing 20% FBS was added to the lower compartment of the chamber. After 24 h, the non-migrated cells in the upper compartment were wiped with a cotton swab, and the migrated cells were washed with PBS three times, then fixed with 4% paraformaldehyde at room temperature for 20 min. After drying, the cells were stained with 0.5% crystal violet for 20 min, then washed and dried. Finally, four visual fields were randomly selected under the microscope, and the number of migrated cells were counted using ImageJ software. The experiment was repeated three times and the average number of migrated cells was calculated.

### Apoptosis assay

Control and si-ASS1-transfected HGC-27 and MKN28 cells were stained with Annexin V-FITC/PI (Lianke, CHINA) after 48 h transfection, and analyzed by flow cytometry.

### Statistical analysis

Statistical analysis was carried out using SPSS 18.0. Continuous data were presented as “x ± s” and compared using Student’s *t*-test. Count data were expressed as rate and analyzed using the chi-squared test. The Kaplan-Meier method and Log-rank test were used to compare the survival rate. Initially, univariable Cox analysis was employed to examine the association between pathological parameters and patient prognosis. Subsequently, multivariable Cox analysis incorporating significant variables in the univariable analysis was performed with a stepwise forward LR method for variable selection. *p* < 0.05 indicates statistical significance.

## Results

### Association between tumor location and clinicopathological features

Tumor size and tumor location were found to be closely associated (*p* < 0.05). Furthermore, a correlation between tumor location and pathological stage was found (*p* = 0.097). No significant correlations were found between tumor location and age, sex, histological type, differentiation degree, infiltration depth, lymph node metastasis, or distant metastasis (all *p* > 0.05) (Table 1).

**Table 1:** Clinicopathological correlations of tumor location and parameters in patients with gastric cancer

Parameter	Number of cases	Location				χ2	*p*
		Upper third	Middle third	Lower third	Mix		
Age at diagnosis							
< 60	45	6	11	16	12	1.709	0.635
≥ 60	49	7	10	14	18		
Gender							
Male	68	11	13	24	20	3.629	0.304
Female	26	2	8	6	10		
Tumor size (cm)							
< 5	53	10	11	21	11	8.982	0.030
≥ 5	41	3	10	9	19		
Histologic type							
Adenocarcinoma	85	13	17	29	26	6.046	0.109
Signet ring cell carcinoma	9	0	4	1	4		
Degree of differentiation							
Medium-low/Medium/High	42	4	9	11	18	3.434	0.329
Low	52	9	12	19	12		
Depth of invasion							
T1/T2	16	3	4	7	2	4.396	0.222
T3/T4	78	10	17	23	28		
Lymph node status							
N0/N1/N2	59	11	13	19	16	0.352	0.354
N3	35	2	8	11	14		
Distant metastasis							
M0	90	13	20	28	29	1.037	0.792
M1	4	0	1	2	1		
TNM stage							
Stage I/II	30	8	5	12	5	7.059	0.070
Stage III/IV	64	5	16	18	15		

### Relationship between tumor location and prognosis of gastric cancer patients

**Fig. 1:**
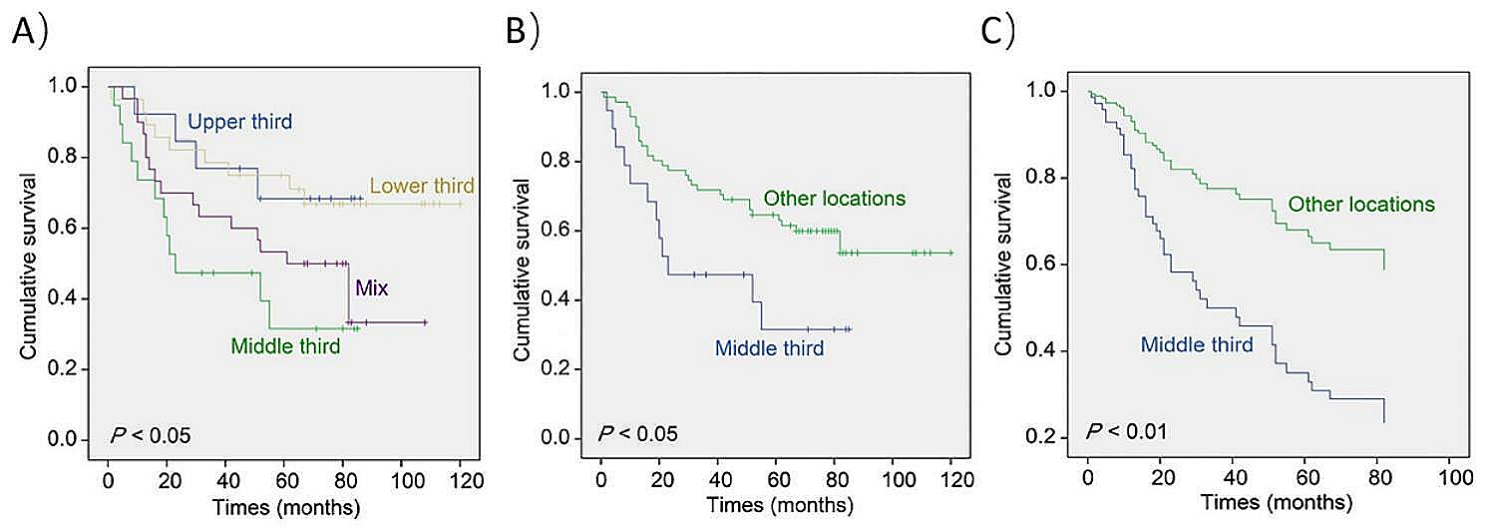
Relationship between tumor location and prognosis of gastric cancer. (**A**, **B**) Kaplan-Meier survival analysis. (**C**) Cox multivariate survival analysis

In univariable Cox analysis, T stage, N stage, M stage, and TNM stage significantly affected the prognosis of gastric cancer patients (all *p* < 0.05) (Table 2), as did the tumor location (*p* = 0.032). Age, sex, tumor size, histological type, and degree of differentiation were not associated with prognosis (all *p* > 0.05). Kaplan-Meier analysis revealed that the average survival time of patients in the Upper third, Middle third, Lower third and Mixed groups were 67.93 ± 7.80, 42.03 ± 7.73, 89.32 ± 8.15 and 61.82 ± 7.40 months, respectively (Fig. 1A). Moreover, primary middle third gastric cancer had a worse prognosis than gastric cancers in other locations (*p* < 0.05) (Fig. 1B).

**Table 2:** Prognostic factors for overall survival of patients with gastric cancer

Parameter	Univariate survival analysis				Multivariate survival analysis		
	HR	95%CI	*p*		HR	95%CI	*p*
Age at diagnosis							
< 60	1.000						
≥ 60	1.628	0.878–3.021	0.122				
Gender							
Male	1.000						
Female	1.116	0.571–2.181	0.748				
Tumor size (cm)							
< 5	1.000						
≥ 5	1.298	0.706–2.387	0.748				
Histologic type							
Adenocarcinoma	1.000						
Signet ring cell carcinoma	1.441	0.348–5.969	0.614				
Degree of differentiation							
Medium-low/Medium/High	1.000						
Low	1.167	0.637–2.140	0.617				
Location							
Other locations	1.000						
Middle third	2.179	1.109–4.281	0.024		2.723	1.334–5.520	0.005
Depth of invasion							
T1/T2	1.000						
T3/T4	3.705	1.142–12.017	0.029		3.357	1.007–11.186	0.049
Lymph node status							
N0/N1/N2	1.000						
N3	5.676	2.953–10.910	< 0.001		5.287	2.723–10.265	< 0.001
Distant metastasis							
M0	1.000						
M1	3.305	1.001–10.914	0.049				
TNM stage							
Stage I/II	1.000						
Stage III/IV	4.214	1.651–10.751	0.003				

HR, Hazard ratio; 95%CI, 95% confidence interval

Multivariable Cox analysis confirmed that primary middle third gastric cancer (HR = 2.723, 95%CI 1.334–5.520) (Fig. 1C), T3/T4 stage (HR = 3.357, 95%CI 1.007–11.186), and N3 stage (HR = 5.287, 95%CI 2.723–10.265) were statistically significant risk factors of overall survival in gastric cancer (Table 2).

### Metabolic profiling

Metabolic profiling showed that in the positive and negative ion modes, the base peak chromatograms of the typical samples in the T vs. N group and Middle vs. Upper/Lower group tended to be consistent, indicating that the analysis system had good repeatability and was reliable (Additional file 4: Figure S1). By calculating the RSD value of the peak area of each precursor molecule in the QC samples, we found that 75.2% of the precursor molecules had < 30% RSD in the positive ion mode, and 64.6% of the precursor molecules had < 30% RSD in the negative ion mode, suggesting that the experimental system was stable and the data were reliable.

### Multivariable analysis of the LC-MS data

**Fig. 2:**
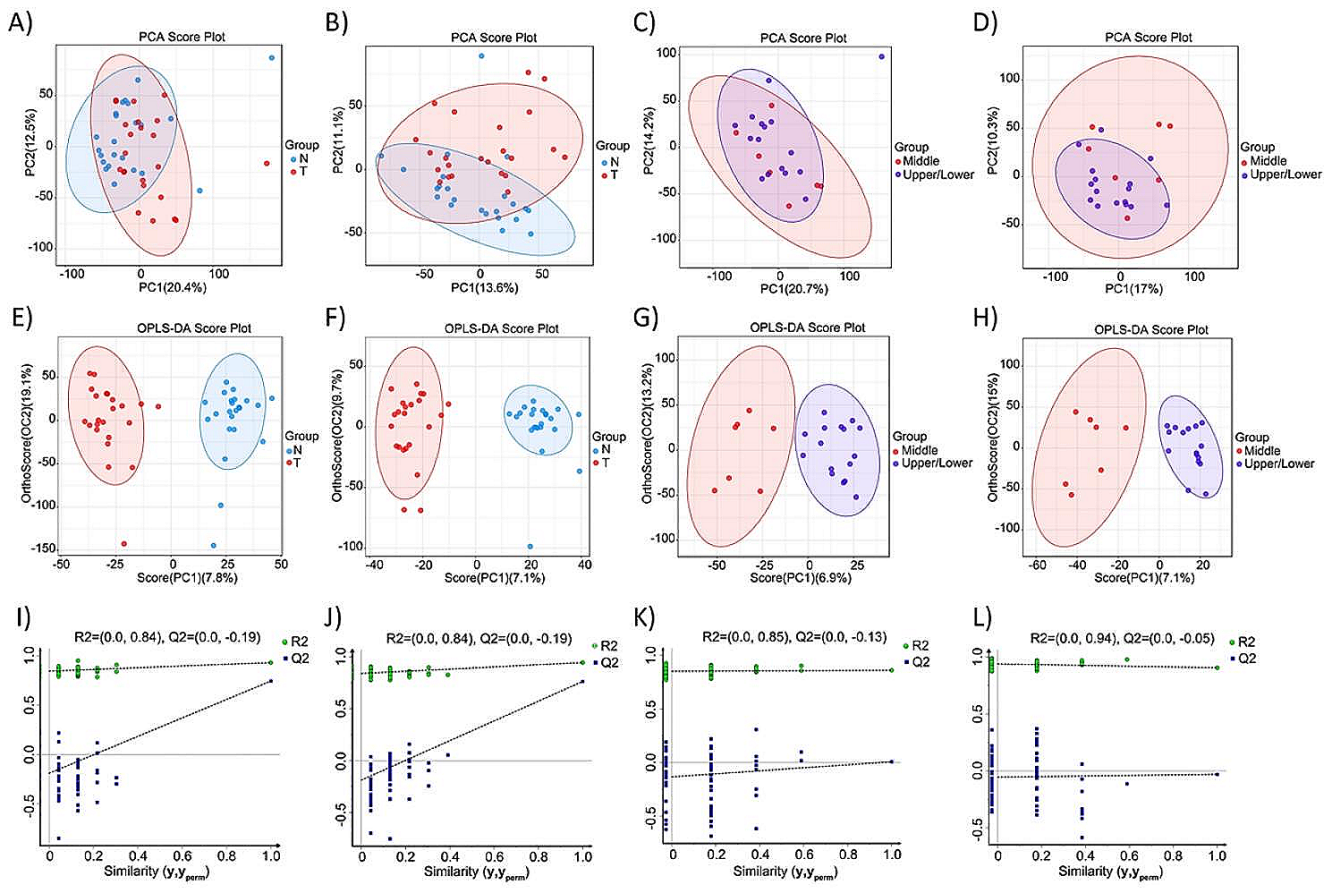
Multivariate statistical analysis of metabolites. PCA scores plot for metabolomics between two groups. (**A**) T vs. N (Positive mode). (**B**) T vs. N (Negative mode). (**C**) Middle vs. Upper/Lower (Positive mode). (**D**) Middle vs. Upper/Lower (Negative mode). Applying OPLS-DA Analysis to evaluate the discriminant ability of the model. OPLS-DA score chart between two groups. (**E**) T vs. N (Positive mode). (**F**) T vs. N (Negative mode). (**G**) Middle vs. Upper/Lower (Positive mode). (**H**) Middle vs. Upper/Lower (Negative mode). Permutation tests for the OPLS-DA models. (**I**) T vs. N (Positive mode). (**J**) T vs. N (Negative mode). (**K**) Middle vs. Upper/Lower (Positive mode). (**L**) Middle vs. Upper/Lower (Negative mode). T, gastric cancer tissues; N, normal tissues; Middle, gastric cancer tissues located in middle third stomach; Upper/Lower, gastric cancer tissues located in upper/lower third stomach; vs., versus

First, we carried out PCA and the extracted principal components were used to classify each group of data through the main new variables (i.e. principal components), and samples with poor repeatability (outlier samples). Abnormal samples were removed. Under the premise that most of the samples were in the 95%CI, the PCA data revealed that in the positive and negative ion modes, the cumulative interpretation rate (R2X (cum)) of the two groups of samples were higher than 0.5 (Additional file 5: Table S3), and thus the two groups were distinguishable (Fig. 2A-D). OPLS-DA was used to evaluate differences between the samples, and the permutation test was used to determine whether the current OPLS-DA model was overfitting. We found that the model had good explanatory and predictive capabilities (Fig. 2E-H) (positive ion model: R2Y = 0.931 in T vs. N group, R2Y = 0.861 in Middle vs. Upper/Lower group; negative ion model: R2Y = 0.944 in T vs. N group, R2Y = 0.904 in Middle vs. Upper/Lower group). The slopes of the lines of the two groups of data in different modes were both > 0, and the Q2 values were all < 0, indicating that the OPLS-DA model was not overfitting (Fig. 2I-L), and therefore a true representation of the differences between the different sample groups.

### Screening and identification of differential metabolites and metabolic pathways

**Fig. 3:**
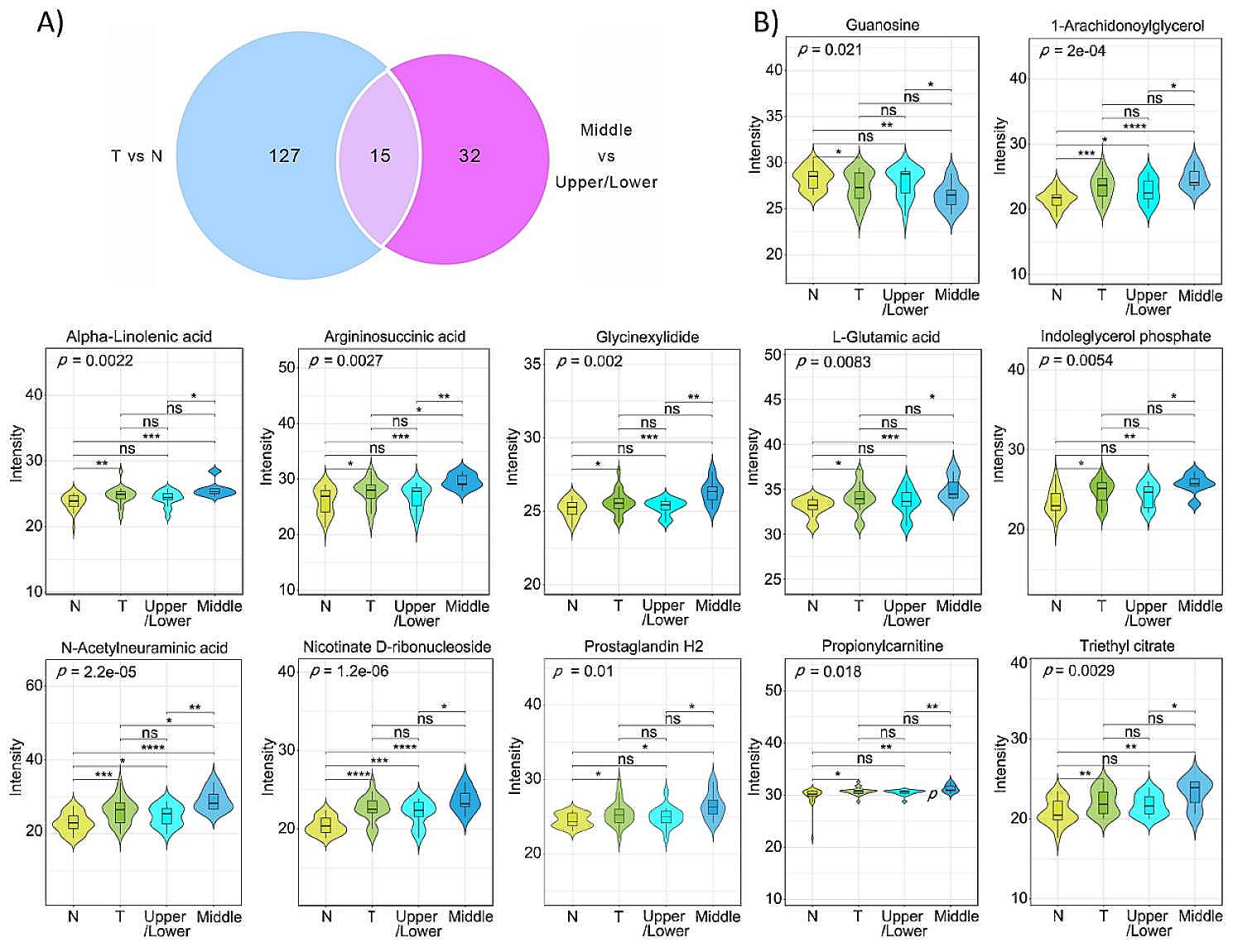
Differentially expressed metabolites in gastric cancer tissue and that in middle third gastric cancer. (**A**) Venn Diagram showed the intersection of the differentially expressed metabolites in gastric cancer tissues and that in middle third gastric cancer. (**B**) The expression level of 12 overlapping metabolites in normal tissues (N), gastric cancer tissues (T), middle third gastric cancer (Middle) and upper/lower third gastric cancer (Upper/Lower). *p < 0.05, **p < 0.01, ***p < 0.001, ns, no significance

The importance of each variable in the model is reflected by the VIP value. For this study, a VIP value of ≥ 1.0 and a *p* value of < 0.05 were used as the cutoff criteria. A total of 142 differential metabolites were identified in the T vs. N group, and 47 in the Middle vs. Upper/Lower group. Fifteen differential metabolites were identified by both comparisons (Fig. 3A; Table 3). Eleven metabolites, including nicotinate D-ribonucleoside, N-acetylneuraminic acid, 1-arachidonoylglycerol, L-glutamic acid, indoleglycerol phosphate, prostaglandin H2, triethyl citrate, alpha-linolenic acid, propionylcarnitine, argininosuccinic acid and glycinexylidide were not only significantly upregulated in gastric cancer tissue compared to normal tissue, but were also significantly increased in the middle third gastric cancer tissue. In contrast, guanosine was significantly downregulated in gastric cancer tissue, particularly in the middle third gastric cancer tissue, indicating the 12 metabolites participated not only in the carcinogenesis of gastric cancer, but were also closely correlated with the poor prognosis of middle third gastric cancer (Fig. 3B).

**Table 3:** List of differentially expressed metabolites

Number	Name	Formula	MZ	RT	T vs. N			Middle vs. Upper/Lower	
					FC	VIP		FC	VIP
1	Nicotinate D-ribonucleoside	C11H14NO6	256.0819	115.2	5.94	2.36		3.13	2.27
2	Phenylacetylglutamine	C13H16N2O4	265.118	456	0.07	2.17		10	1.69
3	(5Z,9E,14Z)-(8xi,11R,12 S)-11,12-epoxy-8-hydroxyicosa-5,9,14-trienoic acid	C20H32O4	317.2122	813.1	1.64	1.78		0.64	1.72
4	(13E)-11a-Hydroxy-9,15-dioxoprost-13-enoic acid	C20H32O5	353.2306	710.1	0.2	1.70		1.75	1.90
5	N-Acetylneuraminic acid	C11H19NO9	292.1025	92	31.63	1.63		27.47	2.27
6	1-Arachidonoylglycerol	C23H38O4	361.2727	837.6	5.24	1.57		3.33	1.66
7	L-Glutamic acid	C5H9NO4	146.043	78.8	2.72	1.47		2.74	2.05
8	Indoleglycerol phosphate	C11H14NO6P	288.0742	90.8	1.92	1.38		2.34	2.15
9	Prostaglandin H2	C20H32O5	351.2179	594.3	3.41	1.30		3.75	1.73
10	Triethyl citrate	C12H20O7	277.128	714.8	2.53	1.27		3.13	1.44
11	Alpha-Linolenic acid	C18H30O2	279.2316	815.7	2.54	1.25		3.31	1.94
12	Guanosine	C10H13N5O5	282.0844	198.6	0.66	1.21		0.38	2.32
13	Propionylcarnitine	C10H19NO4	218.1388	218.6	1.56	1.03		1.88	2.68
14	Argininosuccinic acid	C10H18N4O6	291.129	89.1	2.91	1.00		5.06	2.49
15	Glycinexylidide	C10H14N2O	179.1179	408.1	1.44	1.00		2.18	2.52

T, gastric cancer tissues; N, normal tissues; Middle, gastric cancer tissues located in middle third stomach; Upper/Lower, gastric cancer tissues located in upper/lower third stomach; vs., versus; FC, fold change; VIP, variable importance in projection; RT, retention time; MZ, mass/charge

### Metabolic pathway analysis

**Fig. 4:**
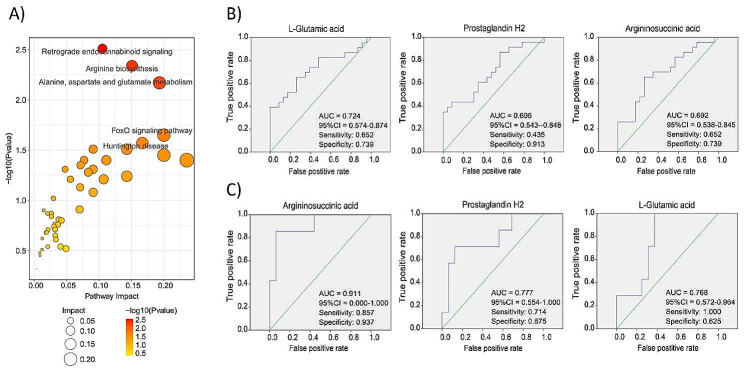
Abnormal metabolic pathways in two groups and ROC curves of related metabolites. (**A**) KEGG pathway of differentially expressed metabolites. (**B**) The ROC curve of 3 differential metabolites discriminating the gastric cancer tissues from normal tissues. (**C**) The ROC curve of 3 differential metabolites discriminating gastric cancer tissues located in middle third stomach from gastric cancer tissues located in upper/lower third stomach. ROC, Multivariate receiver operating characteristic; AUC, area under the curve; 95%CI, 95% confidence interval

Correlations among the metabolites were measured using the Pearson correlation coefficient or Spearman rank correlation coefficient. As shown in Additional file 6: Figure S2, using the KEGG database with a *p* value < 0.05 to screen differential metabolic pathways among various samples, similar pathways were found to be enriched in the T vs. N group and Middle vs. Upper/Lower group (Fig. 4A). The three most notable pathways were retrograde endocannabinoid signaling, arginine biosynthesis and alanine, aspartate and glutamate metabolism. Furthermore, abnormal metabolism of amino acids was found to be one of the key characteristics that distinguished gastric cancer tissue from normal tissue, and middle third gastric cancer from gastric cancer in other locations. Further analysis revealed that the following metabolites were shared by all three pathways, L-glutamic acid, argininosuccinic acid and prostaglandin H2. In addition, receiver operating characteristic (ROC) curve analysis indicated that the area under the curve (AUC) values of these three metabolites were 0.724, 0.692 and 0.696, respectively, for distinguishing gastric cancer from normal tissue (Fig. 4B). Moreover, for differentiating between middle third gastric cancer and gastric cancers in other locations, the corresponding AUC values were 0.768, 0.911 and 0.777, respectively (Fig. 4C).

### Knockdown of ASS1 inhibits the progression of gastric cancer

**Fig. 5:**
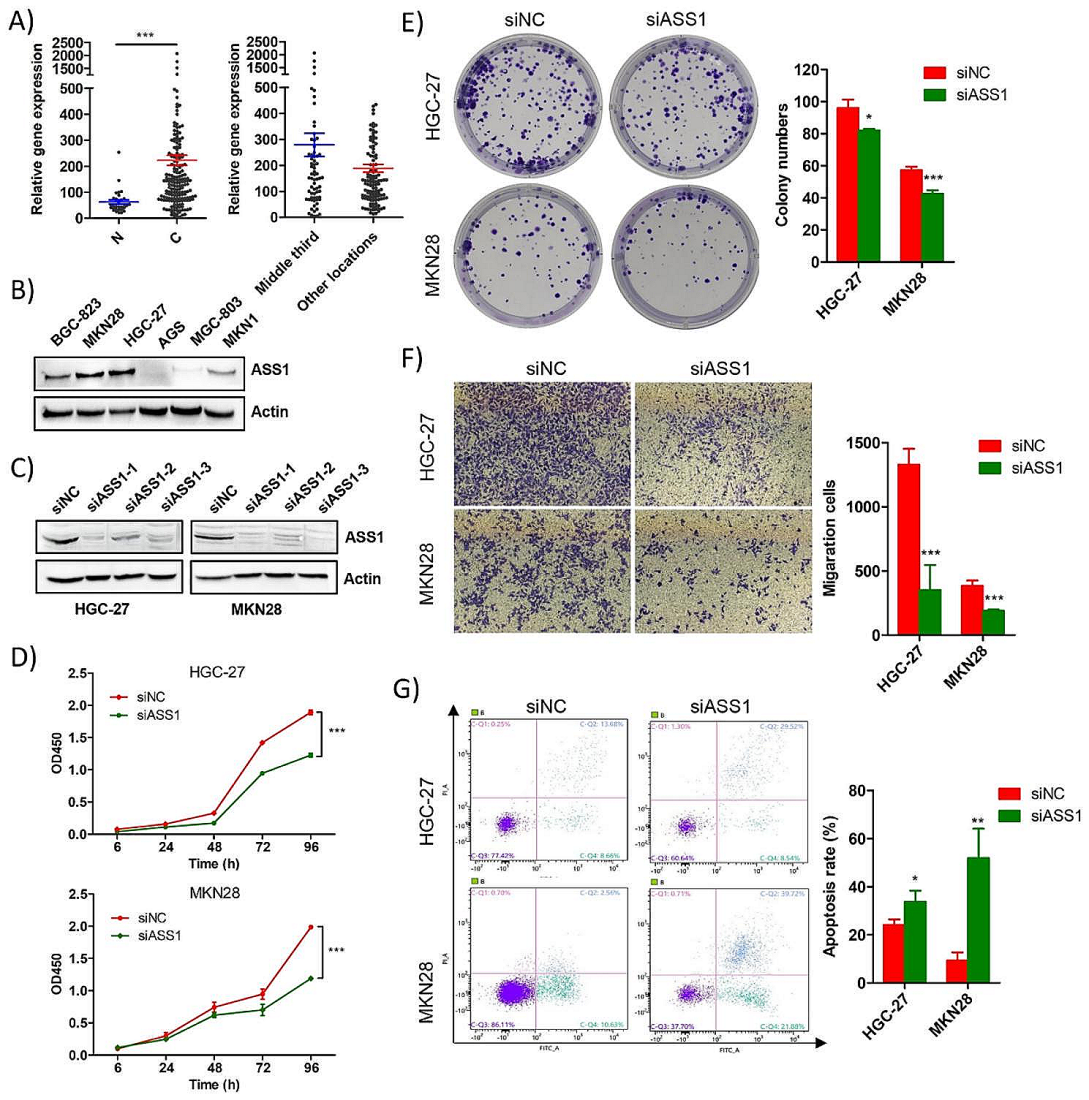
ASS1 was up-regulated in gastric cancer, and knockdown of ASS1 inhibit progression of gastric cancer. (**A**) TCGA data analysis showed that ASS1 was significantly up-regulated in gastric cancer tissues, and also showed an upward trend in middle-third gastric cancer compared to the tumors located in other locations. C, gastric cancer tissues; N, normal tissues. (**B**) Expression level of ASS1 in various gastric cancer cells. (**C**) Western blot successfully confirmed the knockdown of ASS1 in gastric cancer cell lines HGC-27 and MKN28. (**D**) CCK8 assay showed that knockdown of ASS1 significantly suppressed the proliferation of gastric cancer cells. (**E**) Colony formation assay showed that knockdown of ASS1 significantly inhibit the clone forming ability of both gastric cancer cells. (**F**) Transwell assay showed that knockdown of ASS1 significantly inhibit the migratory ability of gastric cancer cells. (**G**) Knockdown of ASS1 significantly promoted the apoptosis of gastric cancer cells. All the data were shown as mean ± SD. *p < 0.05, **p < 0.01, ***p < 0.001

ASS1 is an upstream catalytic enzyme required for the synthesis of argininosuccinic acid, and a key rate-limiting enzyme for arginine synthesis. To determine whether abnormal expression of ASS1 contributed to poor patient prognosis in middle third gastric cancer, we analyzed 197 gastric cancer samples based on their stomach location data, as well as 32 normal tissue samples from the TCGA database. We found that *ASS1* expression was significantly upregulated in gastric cancer samples compared to normal samples (*p* < 0.001). Furthermore, *ASS1* was found to be upregulated in the middle third gastric cancer samples compared to samples from other locations (*p* = 0.059) (Fig. 5A). Western blot analysis revealed that ASS1 expression levels were higher in the gastric cancer cell lines, MKN28 and HGC-27 (Fig. 5B). Knockdown of ASS1 with siRNA led to a significant decrease in the proliferative ability (both *p* < 0.001) (Fig. 5C, D), and the number of colonies in both cell lines of both cell lines (*p* < 0.05, *p* < 0.001, respectively) (Fig. 5E). The migratory abilities of the cells were assessed using the transwell assay. As shown in Fig. 5F, the number of invading HGC-27 and MKN28 cells was significantly decreased following ASS1 knockdown (both *p* < 0.001). Furthermore, ASS1 knockdown resulted in a significant increase in apoptosis in HGC-27 and MKN-28 cells compared to the control group (both *p* < 0.05) (Fig. 5G).

## Discussion

Our study demonstrated that middle third primary gastric cancer had a worse prognosis, and was an independent risk factor for gastric cancer prognosis. Tumor location has been shown to be an important factor that influences the classification of gastric cancer, risk of lymph node metastasis and surgical approaches. The general consensus is that proximal gastric cancer tends to exhibit higher aggressiveness and poorer prognosis than distal gastric cancer [5, 12, 13]. This observation may be attributed to several factors associated with distal gastric cancer, including younger patient age, reduced intraoperative blood loss, relatively lower rate of lymph node metastasis, and a lower proportion of stage IV [12, 13]. Previously, Li et al. investigated the association between tumor location and patient prognosis in 2145 gastric cancer cases, and found that tumors located in the gastric body had a lower 5-year survival rate than tumors located in the gastric fundus, gastric cardia and antrum [14, 15]. In addition, Liu et al. showed that in 198 young gastric cancer patients, those with middle third tumors had a 50.6% overall survival rate, which was significantly lower than those with upper-third or lower-third tumors [15]. Our findings are consistent with these studies and highlight the importance of tumor location in determining patient prognosis.

Next, we found that middle third gastric cancer was associated with increased tumor size, which was consistent with a retrospective study by Li et al. involving 2477 gastric cancer patients [14]. Their study showed that tumors in the gastric body were larger (*p* = 0.009) and associated with lower grade of differentiation and deeper infiltration (T stage) than tumors in the cardia, fundus, and antrum [14]. In another independent cohort study that included gastric cancer patients from South Korea and the United States, the largest tumor size and deepest invasion depth were observed in upper third tumors, while middle third tumors were the second largest in size, and lower third tumors were the smallest [16]. Tumor size has previously been recognized as an independent prognostic factor for gastric cancer [17, 18]. Our study revealed that patients with larger tumors and tumors located in the middle third of the stomach had an elevated risk of poor prognosis.

Using non-targeted metabolomics, we aimed to delineate the characteristic metabolic profiles of gastric cancer at different stomach locations to determine the association between tumor location and tumor size, as well as prognosis. Fifteen differentially expressed metabolites were identified between gastric cancer tissue and normal tissue. These metabolites were enriched in three major pathways including retrograde endocannabinoid signaling, arginine biosynthesis, and alanine, aspartate and glutamate metabolism. An increasing number of studies have indicated a strong association between abnormal amino acid metabolism and the carcinogenesis or progression of gastric cancer [19, 20]. Cancerous cells that lack arginine have been shown to exhibit distinctive traits, including mitochondrial dysfunction, transcriptional reprogramming, and ultimately cell death, and may therefore be the basis for the development of therapeutic approaches that specifically target the synthesis of arginine [21, 22]. Five amino acids including arginine identified by LC-MS analysis were found to be significantly differentially expressed between the plasma of patients with gastric ulcer and gastric cancer, and may therefore act as potential biomarkers for the early detection of gastric cancer [23]. Metabolomics studies based on GC-TOF-MS and UHPLC-QE-M also found dysregulation of arginine metabolism in the tongue coating of patients with gastric precancerous lesions [24]. In addition, a characteristic metabolic panel that included arginine was shown to have high diagnostic efficiency for distinguishing between gastric cancer and superficial gastritis and atrophic gastritis [25]. Together, all these studies suggest that abnormal arginine metabolism may be involved in the carcinogenesis and progression of gastric cancer.

Here, we examined the metabolic profiles of gastric cancer located in the middle third region, as well as other locations. Our findings revealed that the distinctive metabolic phenotype of middle third gastric cancer involved abnormal metabolism of alanine, aspartate, and glutamate, which are closely associated with arginine biosynthesis. Furthermore, our study found that patients with middle third gastric cancer experienced a more unfavorable prognosis. Thus, abnormal arginine metabolism in middle third gastric cancer could potentially influence tumor size in this specific region and subsequently impact patient prognosis.

To further understand the involvement of abnormal arginine metabolism in gastric cancer, we examined the role of ASS1, a key rate-limiting enzyme in this pathway, in gastric cancer cells. ASS1 catalyzes the synthesis of argininosuccinic acid from citrulline and aspartic acid, ultimately leading to the production of arginine via the action of argininosuccinate lyase. Interestingly, we found that ASS1 levels were significantly increased in gastric cancer tissues with a more pronounced elevation observed in middle third gastric cancer. Our findings were consistent with those of Tsai et al., and provided further support for the association between aberrant arginine metabolism and the development of gastric cancer [26]. Finally, we found that knockdown of ASS1 significantly inhibited the proliferation, colony formation, and migration of gastric cancer cells, and promoted apoptosis, further supporting a cancer-promoting role for ASS1 in gastric cancer. The abnormal upregulation of ASS1 in gastric cancer is not only a direct reflection of the association between dysregulation of the arginine synthesis pathway and the carcinogenesis of gastric cancer, but also indirectly corroborates the relationship between abnormal elevation of argininosuccinic acid, and the unfavorable prognosis observed in middle third gastric cancer. Together, these results suggest the potential value of targeting the arginine anabolic pathway in the targeted treatment of gastric cancer.

## Conclusions

In summary, our study described the unique metabolomic characteristics of middle third gastric cancer, and demonstrated that abnormal amino acid metabolism such as arginine synthesis dysregulation is involved in the carcinogenesis of gastric cancer and is associated with the worse prognosis of middle third gastric cancer. Our study provides theoretical basis for the targeted and stratified treatment of gastric cancer.

### Electronic supplementary material

Below is the link to the electronic supplementary material.

**Supplementary Material 1:** The siRNA sequence for *ASS1*

**Supplementary Material 2:** Cumulative interpretation rate (R2X (cum)) of PCA analysis

**Supplementary Material 3:** Sample data

**Supplementary Material 4:** Extraction of metabolites, liquid chromatography-mass spectrometry (LC-MS) analysis, metabolomics data analysis

**Supplementary Material 5:** Typical sample base peak chromatogram

**Supplementary Material 6:** The complete image of the Western blot

**Supplementary Material 7:** Correlation analysis of differential metabolites

## Data Availability

All data generated in this study are available in this article.

## Abbreviations

ASS1: argininosuccinate synthetase 1
FBS: fetal bovine serum
LC-MS: liquid chromatography-mass spectrometry
QC: quality control
ESI: electrospray ionization source
HCD: higher-energy collisional dissociation
RSD: a relative standard deviation
PCA: principal component analysis
PLS-DA: partial least-squares-discriminant analysis
OPLS-DA: orthogonal partial least squares discriminant analysis
VIP: variable influence on projection
TCGA: The Cancer Genome Atlas
ROC: receiver operating characteristic
AUC: area under the curve
