# Supplementary material for: Abnormal arginine synthesis confers worse prognosis in patients with middle third gastric cancer
Source: Cancer Cell Int. 2024 Jan 3;24:6. doi: 10.1186/s12935-023-03200-5 (PMC10765926; doi:10.1186/s12935-023-03200-5)
Supplement: Supplementary file 1 — Supplementary Material 1: The siRNA sequence for ASS1 [file 12935_2023_3200_MOESM1_ESM.docx]

**Additional file 3: Table S2**

Table S2. The siRNA sequence for *ASS1*

| **siRNA** | **Sequence sense（5'-3'）** | **Antisense（5'-3'）** |
| --- | --- | --- |
| siASS1-1 | CCCGCAAACAAGUGGAAAUTT | AUUUCCACUUGUUUGCGGGTT |
| siASS1-2 | CCCACUGUCUCUCUACAAUTT | AUUGUAGAGAGACAGUGGGTT |
| siASS1-3 | CUGGUGUAUACCGGUUUCUTT | AGAAACCGGUAUACACCAGTT |
