# Supplementary material for: Abnormal arginine synthesis confers worse prognosis in patients with middle third gastric cancer
Source: Cancer Cell Int. 2024 Jan 3;24:6. doi: 10.1186/s12935-023-03200-5 (PMC10765926; doi:10.1186/s12935-023-03200-5)
Supplement: Supplementary file 2 — Supplementary Material 2: Cumulative interpretation rate (R2X (cum)) of PCA analysis [file 12935_2023_3200_MOESM2_ESM.docx]

**Additional file 5: Table S3**

Table S3. Cumulative interpretation rate (R2X (cum)) of PCA analysis

| **Comparison** | **Model** | **R2X(cum)** |
| --- | --- | --- |
| T vs. N | positive | 0.525 |
| T vs. N | negative | 0.507 |
| Middle vs. Upper/Lower | positive | 0.506 |
| Middle vs. Upper/Lower | negative | 0.531 |

T, gastric cancer tissues; N, normal tissues; Middle, gastric cancer tissues located in

middle third stomach; Upper/Lower, gastric cancer tissues located in upper/lower

third stomach; vs., versus.
