# Supplementary material for: Abnormal arginine synthesis confers worse prognosis in patients with middle third gastric cancer
Source: Cancer Cell Int. 2024 Jan 3;24:6. doi: 10.1186/s12935-023-03200-5 (PMC10765926; doi:10.1186/s12935-023-03200-5)
Supplement: Supplementary file 3 — Supplementary Material 3: Sample data [file 12935_2023_3200_MOESM3_ESM.docx]

**Additional file 1: Table S1**

Table S1. Sample data

| **Patient** | **Diagnosis** | **Age (year)** | **Gender** | **Location** | **Histologic type** | **Degree of differentiation** | **Depth of invasion (T)** | **Lymph node status (N)** | **Distant metastasis (M)** | **Pathologic stage** | **OS (status)** | **OS (months)** |
| --- | --- | --- | --- | --- | --- | --- | --- | --- | --- | --- | --- | --- |
| 1 | GC | 68 | Male | Upper third | Adenocarcinoma | Low | T4a | N2 | M0 | IIIA | 0 | 72 |
| 2 | GC | 64 | Male | Upper third | Adenocarcinoma | Moderate | T4a | N1 | M0 | IIIA | 0 | 32 |
| 3 | GC | 53 | Male | Upper third | Adenocarcinoma | Moderate/low | T4b | N1 | M0 | IIIB | Lost follow-up | |
| 4 | GC | 62 | Male | Upper third | Adenocarcinoma | Moderate | T4a | N2 | M0 | IIIA | 0 | 5 |
| 5 | GC | 66 | Male | Upper third | Adenocarcinoma | Low | T4a | N3b | M0 | IIIC | 0 | 1 |
| 6 | GC | 60 | Male | Middle third | Adenocarcinoma | Low | T4a | N2 | M0 | IIIA | 0 | 83 |
| 7 | GC | 67 | Male | Middle third | Adenocarcinoma | Moderate/low | T3 | N2 | M0 | IIIA | 0 | 32 |
| 8 | GC | 69 | Male | Middle third | Adenocarcinoma | Moderate/low | T4a | N3b | M0 | IIIC | 1 | 20 |
| 9 | GC | 54 | Male | Middle third | Adenocarcinoma | Low | T3 | N3a | M0 | IIIB | 1 | 23 |
| 10 | GC | 50 | Female | Middle third | Adenocarcinoma | Low | T4b | N3b | M0 | IIIC | 1 | 10 |
| 11 | GC | 67 | Female | Middle third | Adenocarcinoma | Moderate/low | T4a | N2 | M0 | IIIA | Lost follow-up | |
| 12 | GC | 53 | Female | Middle third | Adenocarcinoma | Low | T4a | N3b | M0 | IIIC | 0 |  |
| 13 | GC | 53 | Male | Lower third | Adenocarcinoma | Low | T3 | N3b | M0 | IIIC | 1 | 50 |
| 14 | GC | 56 | Male | Lower third | Adenocarcinoma | Low | T3 | N3a | M0 | IIIB | 1 | 28 |
| 15 | GC | 47 | Male | Lower third | Adenocarcinoma | Low | T4a | N2 | M0 | IIIA | 0 | 36 |
| 16 | GC | 63 | Male | Lower third | Adenocarcinoma | Low | T4a | N2 | M0 | IIIA | 0 | 20 |
| 17 | GC | 66 | Male | Lower third | Adenocarcinoma | Moderate/low | T4a | N3b | M0 | IIIC | 0 | 2 |
| 18 | GC | 58 | Male | Lower third | Adenocarcinoma | Moderate/low | T4a | N3a | M0 | IIIB | 0 | 108 |
| 19 | GC | 44 | Male | Lower third | Adenocarcinoma | Moderate/low | T2 | N3a | M0 | IIIA | 0 | 107 |
| Table S1. Continued. | | | | | | | | | | | | |
| **Patient** | **Diagnosis** | **Age (year)** | **Gender** | **Location** | **Histologic type** | **Degree of differentiation** | **Depth of invasion (T)** | **Lymph node status (N)** | **Distant metastasis (M)** | **Pathologic stage** | **OS (status)** | **OS (months)** |
| 20 | GC | 62 | Male | Lower third | Adenocarcinoma | Low | T3 | N2 | M0 | IIIA | 0 | 70 |
| 21 | GC | 52 | Male | Lower third | Adenocarcinoma | Low | T4b | N3b | M0 | IIIC | 1 | 12 |
| 22 | GC | 53 | Male | Lower third | Adenocarcinoma | Moderate/low | T4a | N3a | M0 | IIIB | 1 | 33 |
| 23 | GC | 52 | Female | Lower third | Adenocarcinoma | Low | T4a | N3a | M0 | IIIB | 1 | 9 |

GC, gastric cancer; OS, overall survival.
