# Supplementary material for: Abnormal arginine synthesis confers worse prognosis in patients with middle third gastric cancer
Source: Cancer Cell Int. 2024 Jan 3;24:6. doi: 10.1186/s12935-023-03200-5 (PMC10765926; doi:10.1186/s12935-023-03200-5)
Supplement: Supplementary file 4 — Supplementary Material 4: Extraction of metabolites, liquid chromatography-mass spectrometry (LC-MS) analysis, metabolomics data analysis [file 12935_2023_3200_MOESM4_ESM.docx]

**Additional file 2: Appendix S1**

**Extraction of metabolites**

An appropriate amount of sample was weighed and transferred into a 2 mL EP tube. Tissue extraction solution (1 mL) containing 75% methanol and chloroform in a 9:1 ratio, and 25% H_2_O, was added to the sample. The sample was ground using a tissue grinder at 50 Hz for 1 min twice, then sonicated at room temperature for 30 min. Samples were incubated on ice for 30 min and centrifuged at 12,000 rpm at 4°C for 10 min. The supernatants were collected, concentrated and dried. Samples were re-dissolved in 2-chloro-L-phenylalanine solution, which was prepared by adding 200 μL of 50% acetonitrile solution. The sample was stored at 4°C for subsequent liquid chromatography–mass spectrometry (LC-MS) analysis. For quality control (QC) sample preparation, a portion of the extracted samples was mixed to produce the QC sample.

**Liquid chromatography-mass spectrometry (LC-MS) analysis**

LC analysis was performed using the Thermo Vanquish LC system (Thermo Fisher Scientific, USA) with an ACQUITY UPLC® HSS T3 column (2.1 × 150 mm, 8 µm). The flow rate was set at 0.25 mL/min, the column temperature was maintained at 40°C, and an injection volume of 2 μL was used. The mobile phase was 0.1% formic acid acetonitrile (C) and 0.1% formic acid water (D) in the positive ion mode, and acetonitrile (A) and 5 mM ammonium formate water (B) in the negative ion mode. The following gradient elution conditions were used: 0~1 min, 2% A/C; 1~9 min, 2%~50% A/C; 9~12 min, 50%~98% A/C; 12~13.5 min, 98% A/C; 13.5~14 min, 98%~2% A/C; and 14~20 min, 2% A/C.

For MS analysis, the Thermo Q Exactive HF-X (Thermo Fisher Scientific, USA) was used with an electrospray ionization source (ESI). Data were collected separately in positive and negative ion modes. The positive ion spray voltage was set to 3.50 kV, while the negative ion spray voltage was set to −2.50 kV. The sheath gas was maintained at 30 arb, and the auxiliary gas at 10 arb. The capillary temperature was set at 325°C. The first-level full scan was conducted at a resolution of 60,000, covering a scanning range of m/z 81 to 1000. The second-level fragmentation was performed using higher-energy collisional dissociation (HCD) with a collision voltage of 30 eV. The second-level resolution was set at 15,000, and dynamic exclusion was implemented to eliminate unnecessary MS/MS data.

**Metabolomics data analysis**

Raw data were converted into the mzXML file format by the ProteoWizard package (v3.0.8789)[1]. The XCMS R package[2] was used for peak detection, peak filtering, and peak alignment processing to obtain a quantitative list of substances. The public databases HMDB[3], MassBank[4], LipidMaps[5], mzCloud[6] and KEGG[7], as well as self-constructed substance databases, were used to identify the substances. The LOESS[8] signal correction method based on QC samples was used for data correction and elimination of systematic errors. During data QC, substances with a relative standard deviation (RSD) > 30% in QC samples were filtered out. The Ropls R package[9] was used to conduct principal component analysis (PCA), partial least-squares-discriminant analysis (PLS-DA) and orthogonal partial least squares discriminant analysis (OPLS-DA) dimensionality reduction on the sample data. Score plots, loading plots and split-plots were drawn. The model was tested for overfitting using the permutation test. Multiple parameters including *p* values, variable influence on projection (VIP) obtained from OPLS-DA dimensionality reduction, and fold change between groups were used to evaluate the influence and interpretation of each metabolite component in classifying and distinguishing samples, thus assisting in the identification of marker metabolites. Metabolites with a *p* value < 0.05 and VIP > 1 were considered to be statistically significant. MetaboAnalyst[10] software was employed to conduct functional pathway enrichment and topology analyses on the differentially metabolized molecules, while KEGG Mapper was used to visualize the enriched pathways.

**References**

1. Smith CA, Want EJ, O'Maille G, Abagyan R, Siuzdak G: **XCMS: processing mass spectrometry data for metabolite profiling using nonlinear peak alignment, matching, and identification**. *Anal Chem* 2006, **78**(3):779-787.

2. Navarro-Reig M, Jaumot J, Garcia-Reiriz A, Tauler R: **Evaluation of changes induced in rice metabolome by Cd and Cu exposure using LC-MS with XCMS and MCR-ALS data analysis strategies**. *Anal Bioanal Chem* 2015, **407**(29):8835-8847.

3. Wishart DS, Tzur D, Knox C, Eisner R, Guo AC, Young N, Cheng D, Jewell K, Arndt D, Sawhney S *et al*: **HMDB: the Human Metabolome Database**. *Nucleic Acids Res* 2007, **35**(Database issue):D521-526.

4. Horai H, Arita M, Kanaya S, Nihei Y, Ikeda T, Suwa K, Ojima Y, Tanaka K, Tanaka S, Aoshima K *et al*: **MassBank: a public repository for sharing mass spectral data for life sciences**. *J Mass Spectrom* 2010, **45**(7):703-714.

5. Sud M, Fahy E, Cotter D, Brown A, Dennis EA, Glass CK, Merrill AH, Jr., Murphy RC, Raetz CR, Russell DW *et al*: **LMSD: LIPID MAPS structure database**. *Nucleic Acids Res* 2007, **35**(Database issue):D527-532.

6. Abdelrazig S, Safo L, Rance GA, Fay MW, Theodosiou E, Topham PD, Kim DH, Fernandez-Castane A: **Metabolic characterisation of Magnetospirillum gryphiswaldense MSR-1 using LC-MS-based metabolite profiling**. *RSC Adv* 2020, **10**(54):32548-32560.

7. Ogata H, Goto S, Sato K, Fujibuchi W, Bono H, Kanehisa M: **KEGG: Kyoto Encyclopedia of Genes and Genomes**. *Nucleic Acids Res* 1999, **27**(1):29-34.

8. Gagnebin Y, Tonoli D, Lescuyer P, Ponte B, de Seigneux S, Martin PY, Schappler J, Boccard J, Rudaz S: **Metabolomic analysis of urine samples by UHPLC-QTOF-MS: Impact of normalization strategies**. *Anal Chim Acta* 2017, **955**:27-35.

9. Thevenot EA, Roux A, Xu Y, Ezan E, Junot C: **Analysis of the Human Adult Urinary Metabolome Variations with Age, Body Mass Index, and Gender by Implementing a Comprehensive Workflow for Univariate and OPLS Statistical Analyses**. *J Proteome Res* 2015, **14**(8):3322-3335.

10. Xia J, Wishart DS: **Web-based inference of biological patterns, functions and pathways from metabolomic data using MetaboAnalyst**. *Nat Protoc* 2011, **6**(6):743-760.
