# Supplementary material for: Abnormal arginine synthesis confers worse prognosis in patients with middle third gastric cancer
Source: Cancer Cell Int. 2024 Jan 3;24:6. doi: 10.1186/s12935-023-03200-5 (PMC10765926; doi:10.1186/s12935-023-03200-5)
Supplement: Supplementary file 5 — Supplementary Material 5: Typical sample base peak chromatogram [file 12935_2023_3200_MOESM5_ESM.docx]

**Additional file 4: Figure S1**


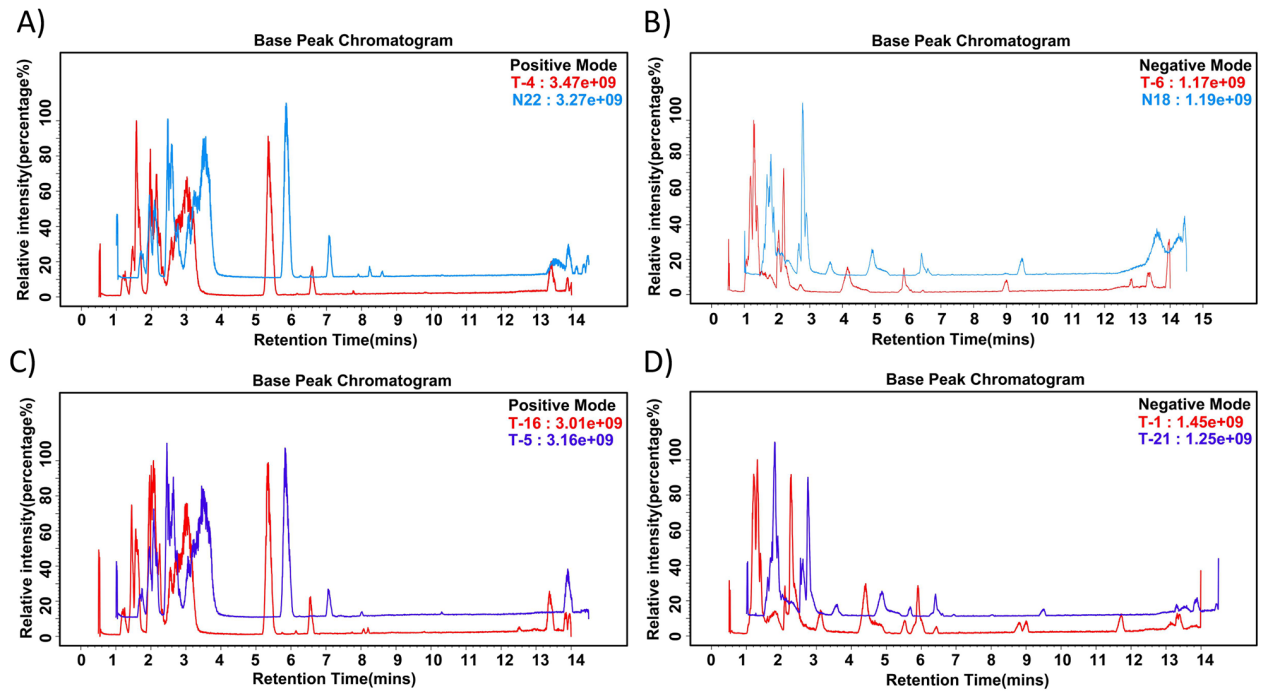


Fig. S1 Typical sample base peak chromatogram. (A) T vs. N (Positive mode). (B) T vs. N (Negative mode). (C) Middle vs. Upper/Lower (Positive mode). (D) Middle vs. Upper/Lower (Negative mode). T, gastric cancer tissues; N, normal tissues; Middle, gastric cancer tissues located in middle third stomach; Upper/Lower, gastric cancer tissues located in upper/lower third stomach; vs., versus.
