# Supplementary material for: Abnormal arginine synthesis confers worse prognosis in patients with middle third gastric cancer
Source: Cancer Cell Int. 2024 Jan 3;24:6. doi: 10.1186/s12935-023-03200-5 (PMC10765926; doi:10.1186/s12935-023-03200-5)
Supplement: Supplementary file 6 — Supplementary Material 6: The complete image of the Western blot [file 12935_2023_3200_MOESM6_ESM.docx]

**Additional file 7: Figure S3**


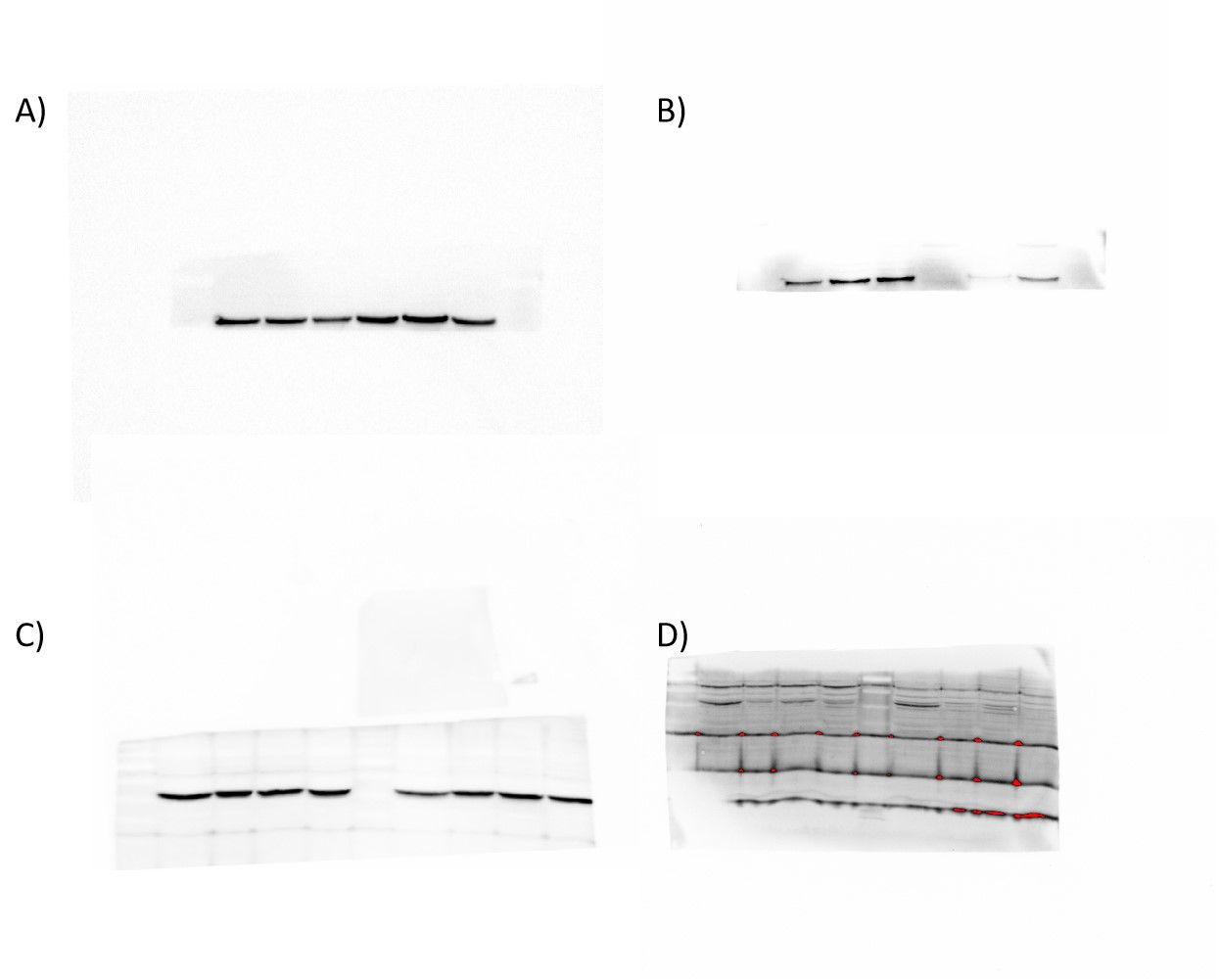


**Fig. S3 The complete image of the Western blot for the Figures 5. A. t**he uncropped blot for fig. 5B (Actin). **B.** the uncropped blot for fig. 5B (ASS1). **C.** the uncropped blot for fig. 5C (Actin). **D.** the uncropped blot for fig. 5C (ASS1).
