# Supplementary material for: Abnormal arginine synthesis confers worse prognosis in patients with middle third gastric cancer
Source: Cancer Cell Int. 2024 Jan 3;24:6. doi: 10.1186/s12935-023-03200-5 (PMC10765926; doi:10.1186/s12935-023-03200-5)
Supplement: Supplementary file 7 — Supplementary Material 7: Correlation analysis of differential metabolites [file 12935_2023_3200_MOESM7_ESM.docx]

**Additional file 6: Figure S2**

**Additional file**


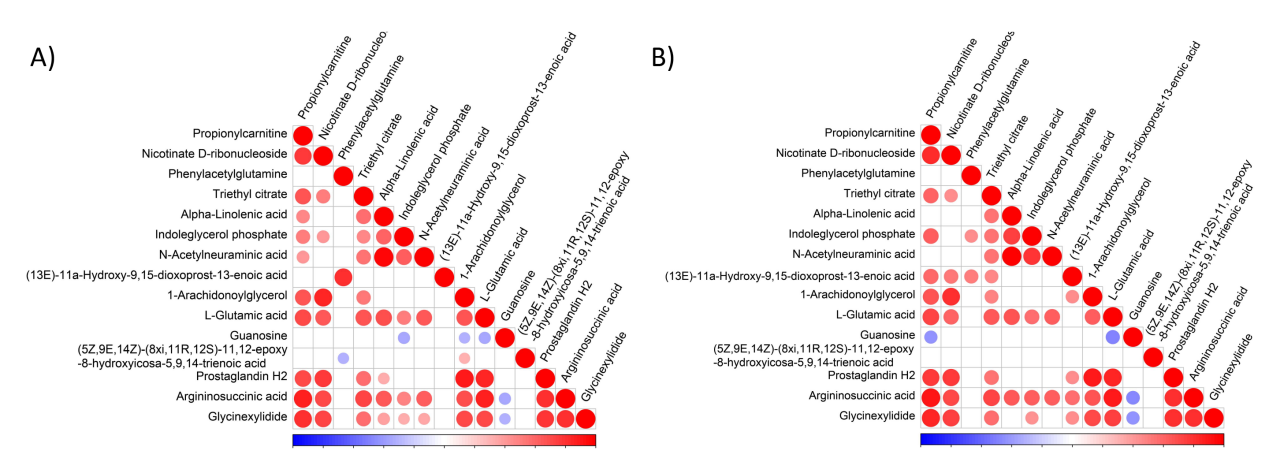


Fig. S2 Correlation analysis of differential metabolites. (A) T vs. N. (B) Middle vs. Upper/Lower. T, gastric cancer tissues; N, normal tissues; Middle, gastric cancer tissues located in middle third stomach; Upper/Lower, gastric cancer tissues located in upper/lower third stomach; vs., versus.
